# Key Clinical Factors Predicting Adipokine and Oxidative Stress Marker Concentrations among Normal, Overweight and Obese Pregnant Women Using Artificial Neural Networks

**DOI:** 10.3390/ijms19010086

**Published:** 2017-12-28

**Authors:** Mario Solis-Paredes, Guadalupe Estrada-Gutierrez, Otilia Perichart-Perera, Araceli Montoya-Estrada, Mario Guzmán-Huerta, Héctor Borboa-Olivares, Eyerahi Bravo-Flores, Arturo Cardona-Pérez, Veronica Zaga-Clavellina, Ethel Garcia-Latorre, Gabriela Gonzalez-Perez, José Alfredo Hernández-Pérez, Claudine Irles

**Affiliations:** 1 Department of Human Genetics and Genomics, Instituto Nacional de Perinatología Isidro Espinosa de los Reyes, Mexico City 11000, Mexico; msolis_83@yahoo.com.mx; 2 Posgrado en Ciencias Químico-Biológicas, Escuela Nacional de Ciencias Biológicas, Instituto Politécnico Nacional, Mexico City 11340, Mexico; ethelagarcia@hotmail.com; 3 Research Division, Instituto Nacional de Perinatología Isidro Espinosa de los Reyes, Mexico City 11000, Mexico; gpestrad@gmail.com (G.E.-G.); mguzmanhuerta@yahoo.com.mx (M.G.-H.); acardonadr@hotmail.com (A.C.-P.); 4 Department of Nutrition and Bioprogramming, Instituto Nacional de Perinatología Isidro Espinosa de los Reyes, Mexico City 11000, Mexico; oti_perichart@yahoo.com (O.P.-P.); h_borboa1@yahoo.com (H.B.-O.); 5 Department of Inmunobiochemistry, Instituto Nacional de Perinatología Isidro Espinosa de los Reyes, Mexico City 11000, Mexico; ara_mones@hotmail.com (A.M.-E.); eyerahiqfb@yahoo.com.mx (E.B.-F.); v.zagaclavellina@gmail.com (V.Z.-C.); 6 Posgrado en Ciencias Biológicas, Universidad Nacional Autónoma de Mexico, Mexico City 04510, Mexico; 7 Department of Physiology and Cellular Development, Instituto Nacional de Perinatología Isidro Espinosa de los Reyes, Mexico City 11000, Mexico; gonzalezperez.gabriela@gmail.com; 8 Centro de Investigación en Ingeniería y Ciencias Aplicadas-Instituto de Investigación en Ciencias Básicas y Aplicadas (CIICAp-IICBA), Universidad Autónoma de Morelos, Cuernavaca 62209, Mexico; alfredo@uaem.mx

**Keywords:** artificial neural networks, pregnancy, oxidative stress markers, adipokines, obesity

## Abstract

Maternal obesity has been related to adverse neonatal outcomes and fetal programming. Oxidative stress and adipokines are potential biomarkers in such pregnancies; thus, the measurement of these molecules has been considered critical. Therefore, we developed artificial neural network (ANN) models based on maternal weight status and clinical data to predict reliable maternal blood concentrations of these biomarkers at the end of pregnancy. Adipokines (adiponectin, leptin, and resistin), and DNA, lipid and protein oxidative markers (8-oxo-2′-deoxyguanosine, malondialdehyde and carbonylated proteins, respectively) were assessed in blood of normal weight, overweight and obese women in the third trimester of pregnancy. A Back-propagation algorithm was used to train ANN models with four input variables (age, pre-gestational body mass index (p-BMI), weight status and gestational age). ANN models were able to accurately predict all biomarkers with regression coefficients greater than R^2^ = 0.945. P-BMI was the most significant variable for estimating adiponectin and carbonylated proteins concentrations (37%), while gestational age was the most relevant variable to predict resistin and malondialdehyde (34%). Age, gestational age and p-BMI had the same significance for leptin values. Finally, for 8-oxo-2′-deoxyguanosine prediction, the most significant variable was age (37%). These models become relevant to improve clinical and nutrition interventions in prenatal care.

## 1. Introduction

Obesity during pregnancy is associated with adverse maternal and neonatal outcomes, and with a higher risk of developing cardiovascular and metabolic diseases in childhood and adult stages. Maternal programming of disease has been widely described [1,2,3,4,5,6,7,8,9]. In particular, obesity programming of the offspring has been postulated to occur periconceptionally and at the end of pregnancy, when an increase in lipid and leptin expression is observed (reviewed by [4]). Maternal obesity is linked to dysregulation of adipose tissue (AT) metabolism [8], and an imbalance in the pro-oxidant-antioxidant system with an increase of reactive oxygen species (ROS) that results in oxidative stress [10,11,12,13]. ROS alter different cellular components such as proteins, lipids and DNA, generating oxidized biomolecules that can be used as biomarkers of oxidative stress. Carbonylated proteins (CP), malondialdehyde (MDA) and the oxidized base 8-oxo-2′-deoxyguanosine (8-oxodG) are indicators of protein oxidation, lipid peroxidation and DNA oxidation, respectively [13].

Several studies in pregnancy have linked increased oxidative stress with lower birth weight [14,15], impaired growth, and higher adiposity in infants [16]. Moreover, maternal AT secretes adipokines such as leptin, adiponectin and resistin [17,18], which have been implicated in metabolic control and fetal programming of adiposity in newborns and infants [19,20,21,22,23]. Thus, quantification of maternal adipokines and oxidized biomolecules could be used as potential biomarkers of perinatal and infant health outcomes [24,25]. Accordingly, prenatal mathematical modeling to predict these biomarkers during pregnancy without blood sampling becomes critical to implement strategies to improve maternal and neonatal outcomes. Artificial Intelligence, particularly artificial neural network (ANN), is a powerful and rigorous tool for the analysis of non-linear and highly complex relationships that learns from all data including experimental and clinical variables, by allowing a very accurate estimation of parameters [26,27]. ANN has been applied in the perinatal field for diagnosis, data mining and clinical decision [28,29,30,31,32]. However, currently no mathematical models have been reported that predict maternal blood biomarkers from clinical and anthropometric data. 

Consequently, the aim of this study was to develop and validate ANN models to predict blood concentrations of adipokines and oxidative stress biomarkers at the end of pregnancy based on pre-gestational weight status and clinical data. Secondly, we calculated the relative importance of these factors in estimating the biochemical values. The third trimester of pregnancy was chosen for the estimation of maternal biomarkers, since it is considered a critical window for developmental programming by maternal obesity, distinct from early pregnancy [4].

## 2. Results

We studied pregnant women during the last trimester of pregnancy (normal weight *n* = 25, overweight *n* = 21, obesity *n* = 22). Mean GA when biochemical measurements were done was 35.2 ± 3.3 weeks. Table 1 shows maternal anthropometric and biochemical data by BMI classification. Normal weight women were younger compared to obese women and had significantly higher concentrations of adiponectin and resistin together with lower MDA and CP levels (*p* < 0.05). 

In this study, mathematical models with neural networks were developed to: (1) predict the concentration of either leptin, adiponectin, resistin, 8-oxodG, MDA and CP in maternal blood at the third trimester of pregnancy (output variable) through a simple equation based on four input variables: pre-gestational maternal age, p-BMI, weight status classification and gestational age at which the sample was taken; and (2) to obtain the most critical pre-gestational parameters influencing the predicted biomarkers.

**Figure 1:**
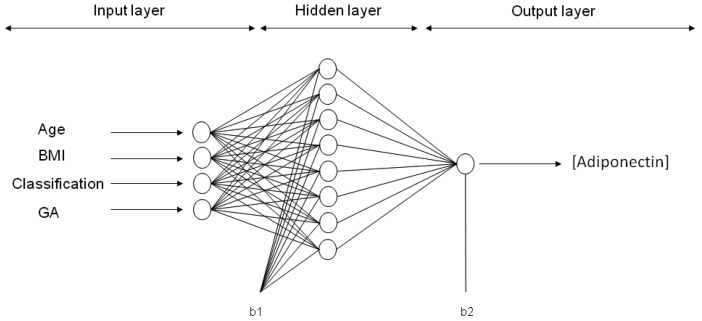
Maternal artificial neural network model. Shown is a representative prediction model for maternal blood adiponectin concentration involving four input variables (maternal age, p-BMI, weight status classification and gestational age), eight neurons in the hidden layer and one output variable (adiponectin concentration).

ANN neurons are organized into multiple connected layers to predict a response. The chosen architecture of the model was an input layer, a hidden layer and an output layer, trained and tested by a back-propagation algorithm (BPNN), as previously described [29]. The percentage of the experimental database (input and output variables) for training and validation was defined. For each model, from one to several neurons were applied in the hidden layer until the minimum root mean square error (RMSE) was obtained between experimental data and predicted values from the neural network (Figure 1). The Levenberg–Marquardt algorithm was chosen for training the model by changing the weights and biases to get the lowest value for the RMSE and being careful to avoid over fitting. The ANN was developed by software toolbox Matlab^®^.

After running distinct conditions, the hyperbolic tangential (TANSIG) transfer function presented the best performance in the hidden layer for all models. In the output layer, the Log-sigmoid (LOGSIG) transfer function was applied for adiponectin, resistin, leptin, and 8-oxodG, while the linear (PURELIN) transfer function was used for MDA and CP.

**Figure 2:**
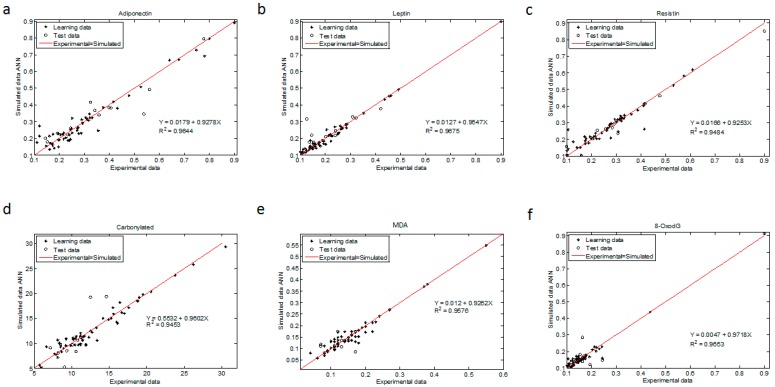
Experimental vs. ANN-simulated values for blood adipokine and oxidative marker concentrations. Scatter plots of: (**a**) adiponectin; (**b**) leptin; (**c**) resistin; (**d**) carbonylated proteins; (**e**) MDA; and (**f**) 8-oxodG maternal levels. Red lines indicate the linear regression model on scatter points. Open circles and closed diamonds depict experimental data and learning data, respectively.

Six ANN models were trained with maternal input variables (age, p-BMI, weight status and GA) to predict the output variable: Either adiponectin, leptin, resistin, CP, MDA or 8-oxodG concentration in maternal blood at the third trimester of pregnancy. After applying 30,000 runs (with 1000 epochs in each model) in the hidden layer (1–9 neurons), the best network architecture performances were found for each adipokine or oxidative stress marker estimation. For ANN models predicting the values of adiponectin, leptin and CP, the final architecture was 4-8-1 (four input variables, eight neurons in the hidden layer, and one neuron in the output layer (biomarker concentration)). The final topology for the MDA model was 4-6-1, while, for resistin and 8-oxodG models, the best performance was 4-9-1. The representative neural architecture for the prediction of adiponectin concentration is shown in Figure 2, whereas the weights, biases and equations for the prediction of all adipokines and oxidative stress marker concentrations are reported in Materials and Methods and Appendix A (Table A1, Table A2, Table A3, Table A4, Table A5 and Table A6).

**Figure 3:**
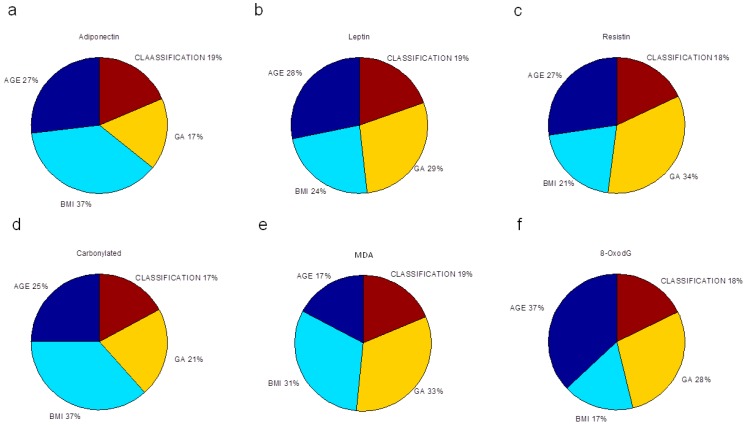
Sensitivity analysis. Percentage of mathematical significance of the four pre-gestational input variables (maternal age, p-BMI, weight status classification and gestational age) in maternal ANN models for: (**a**) adiponectin; (**b**) leptin; (**c**) resistin; (**d**) carbonylated proteins; (**e**) MDA; and (**f**) 8-oxodG concentrations.

The regression coefficient for all ANN models were above 0.945 (R^2^ > 0.9644 for adiponectin, R^2^ > 0.9675 for leptin, R^2^ > 0.9484 for resistin, R^2^ > 0.9453 for CP, R^2^ > 0.9576 for MDA and R^2^ > 0.9653 for 8-oxodG (Figure 3)). The statistical test from these plots showed that the upper and lower values of the slope and intercept included 1 and contained 0, respectively, with a 99.9% confidence level for all determinations (Materials and Methods and Table A7 and Table A8 in Appendix A).

Finally, we evaluated the relative importance of pre-gestational variables in the neural network modeling of adipokine and oxidative stress marker concentrations, depicted as the percentage of quantitative significance (Figure 3). The sensitivity analysis is based on the ANN weight matrix and the Garson equation [33] (Materials and Methods). All maternal factors were essential in estimating the studied biochemical markers. P-BMI was the most important predictor of adiponectin followed by age. For leptin prediction, age, GA and p-BMI value had the same importance. The most relevant factor in forecasting resistin was GA; other predictor factors were age and p-BMI value. For oxidative markers, GA and p-BMI estimated MDA, while CP were predominantly predicted by p-BMI, followed by age and GA. Finally, for 8-oxodG, the meaningful variable was age, then GA and p-BMI. Weight status was the weakest predictor in all models.

## 3. Discussion

Nutrition and metabolic changes occur during pregnancy to promote optimal fetal growth and development. The presence of obesity in pregnancy is associated with nutrient and hormonal imbalances, and with inflammation [34]. Altered leptin, adiponectin, and oxidative stress markers have been documented in pregnant women with obesity [25,35], and have been associated with adverse perinatal outcomes [36,37,38]. The early prediction of alterations in these markers at the end of pregnancy is very relevant, considering the high prevalence of overweight/obesity in women of reproductive age in many countries [39].

ANN is a tool that allowed the estimation of these biochemical markers with anthropometric and clinical variables that are generally used in clinical practice. We present six ANN models that accurately predict third trimester maternal concentrations of adipokines (adiponectin, leptin, and resistin) and DNA, lipid and protein oxidative damage markers (8-oxodG, MDA and CP, respectively). Regression coefficients between the experimental and predicted values for all determinations were superior to R^2^ = 0.945.

For adipokines, the model correctly estimated higher leptin concentrations together with lower adiponectin and resistin values in obese mothers in comparison with normal and overweight pregnant women, a finding that has been reported before [25,40]. In particular, the ANN-predicted adiponectin values (that learned from the experimental data) were similar to those found in the literature [41]. 

We found that leptin prediction was equally dependent on GA, p-BMI and maternal age. This is in agreement with the literature where leptin concentration increases with gestational age in normal pregnancies, mainly produced by adipose tissue, placenta, skeletal muscle and mammary gland (reviewed by [42]). Many studies also have shown increased leptin concentrations with higher p-BMI and in overweight/obese women [35,43,44].

Maternal adiponectin has been inversely related with BMI [45], GA and positively associated with maternal age [46,47]. The ANN adiponectin model derived from this study was able to predict these associations too. P-BMI was the most important parameter in estimating adiponectin and carbonylated proteins. 

Pregnancy per se is an oxidative stress condition due to a higher oxygen demand [48]. Protein carbonylation is caused by the direct attack of free radicals, by interaction with transition metals, by glycation or by adduct formation with final lipoperoxidation products (MDA) (reviewed by [49]). In our models, CP and MDA estimated values were significantly increased in obese pregnancies compared to normal weight mothers, suggesting an increased oxidative damage in the latter and in line with the literature [24,50]. In this work, a higher p-BMI was related to decreased adiponectin and increased MDA concentrations, in agreement with a negative correlation between adiponectin and oxidative lipid damage during pregnancy [51].

Gestational age at sample collection was a relevant factor (34%) for resistin and MDA predictions, suggesting that resistin and MDA levels are greatly influenced by gestational age at the end of pregnancy, despite differences in maternal BMI. These results could be explained by the resistin-dependent insulin resistance that is increased during pregnancy and is modulated in part by a higher glucose transporter 1 (GLUT-1) expression in trophoblast cells induced by resistin [52]. 

Concerning oxidative damage markers, there is a physiological increase associated with women’s aging [53] as well as with advanced gestational age in normal pregnancies [54]. Interestingly, for 8-oxodG, the meaningful variable was maternal age (37%). The link between oxidative stress and aging has been discussed in recent years. In human clinical cohorts, increased 8-oxodG levels in muscle and leukocyte DNA were observed with increasing age [55]. No studies have been reported in pregnancy. Investigating changes in this DNA oxidative marker during pregnancy in different age groups is pending.

### Study Limitations and Strengths

A limitation of this study is the sample size (*n* = 68), however, the model accurately estimated blood concentrations with regression coefficients with values >0.9. Furthermore, other ANN studies have shown validated results with smaller samples [30]. It is important to mention that these ANN models will predict accurately adipokines and oxidative stress markers within the range in which they learn, for example healthy women with singleton pregnancy, maternal p-BMI range between 18.6 and 48.3 kg/m^2^.

## 4. Materials and Methods 

### 4.1. Study Design and Ethical Approval

This research was approved by the IRB of the Instituto Nacional de Perinatología Isidro Espinosa de los Reyes (register 3300-11402-01-575-17), and was conducted according to the relevant national regulations and the Helsinki Declaration with its later amendments (1985). Participation was voluntary and all women who agreed to participate signed an informed consent.

### 4.2. Characteristics of the Population

Healthy women with singleton pregnancy, and with blood sample taken during the third trimester of gestation (28–40 weeks of gestation) were enrolled in the study (*n* = 68). Samples were selected by convenience and stratified by pregestational body mass index (p-BMI). Gestational age (GA) was determined using the last menstrual period date; if GA with this method differed significantly from first trimester ultrasound measurement, then the latter was used. Women with multiple pregnancies, Type 2 Diabetes Mellitus or gestational diabetes mellitus, chronic or gestational hypertension, renal or autoimmune disease, intrauterine fetal growth restriction, fetal structural abnormalities or drug intake that affects metabolism and/or inflammation (metformin, steroids, insulin, and antihypertensives, among others) were excluded. 

### 4.3. Anthropometry

Pre-gestational weight was self-reported. Stature (cm) was measured with a stadiometer (SECA 220, Hamburg, Germany) by trained personnel. P-BMI was calculated using the following formula: Weight/stature^2^. BMI classification was done according to the World Health Organization criteria, where a p-BMI > 18.5 was classified as normal weight, p-BMI > 25 as overweight, and >30 as obesity. 

### 4.4. Biochemical Markers

Maternal blood samples were collected in the fasting state, in Vacutainer tubes (Becton-Dickinson, Franklin Lakes, NJ, USA) and centrifuged at 4 °C for 15 min at 1000× *g*. The serum and plasma samples were stored at −80 °C until the assays were performed.

Serum glucose, adiponectin, leptin, resistin, and insulin concentrations were measured by ELISA commercial kits, as previously described [20]. Adiponectin, leptin and resistin (R & D Systems Inc., Minneapolis, MN, USA) had the following sensitivities: 0.891 ng/mL, 7.8 ng/mL and 0.055 ng/mL, respectively; and assay ranges: 3.9–250 ng/mL; 15.6–1000 pg/mL and 0.2–10 ng/mL, respectively. Insulin (Sigma-Aldrich, St. Louis, MO, USA) and glucose (DiaSys Diagnostic Systems GmbH, Holzheim, Germany) sensitivities were: 2.15 pmol/L, and 1 mg/dL, respectively; and assay ranges were: 15.6–500 pmol/mL and 1–400 mg/dL, respectively.

Homeostatic model assessment (HOMA) index was calculated according to [56]. Oxidative DNA damage level was measured using an 8-oxodG ELISA kit (TREVIGEN, Gaithersburg, MD, USA) with a sensitivity and assay range of 0.57 ng/mL and 0.89–56.7 ng/mL, respectively. Plasma malondialdehyde (MDA) was quantified as described by Gerard et al. [57] with 1-methyl-2-phenylindole (Sigma-Aldrich, St. Louis, MO, USA) as a standard. Sensitivity for MDA determination was 17 ng/mL and assay range of 0.27–2000 ng/mL. Protein damage was evaluated by plasma carbonyl group content, which was determined with 2,4-dinitrophenylhydrazine (DNPH), and measured according to Amici et al. [58]. Assay range and sensitivity for CP evaluation were: 1–10 mg/mL and 1.5 nmol/mg, respectively.

### 4.5. Statistical Analysis

Descriptive analysis (data distribution, frequencies) were done. One-way ANOVA with DMS post hoc test was used to analyze differences by p-BMI categories (normal weight, overweight or obese). Data are expressed as mean ± SEM, and *p* values ≤ 0.05 were considered statistically significant. Statistical analysis was performed using the IBM SPSS v20.0 software (IBM Corporation, Armonk, NY, USA).

### 4.6. ANN (Learning, Testing and Validation)

**Figure 4:**
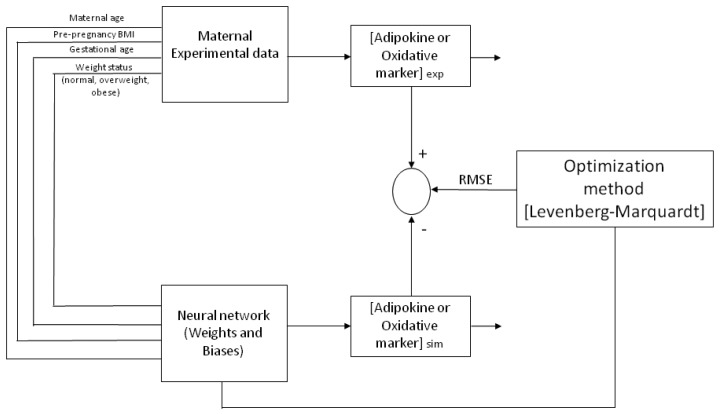
A representative network architecture of the maternal artificial neural network (ANN) model. The learning procedure used by ANN for the prediction at the third trimester of pregnancy of adipokine or oxidative stress marker concentrations in maternal blood (from 4 gestational variables: maternal age, p-BMI, weight status classification and gestational age), trained by the Levenberg–Marquardt optimization algorithm. The same architecture was utilized for adiponectin, leptin, resistin, carbonylated proteins, MDA and 8-oxodG values estimation. + and – indicate changing the weights and biases values to obtain the smallest error between exp and sim.

An artificial neural network utilizes nodes (neurons) connected between each other in distinct layers, their relationship being defined by weights (*Wi*, *Wo*) and biases (*b*1, *b*2) that are obtained by iterations with the ANN algorithm. Figure 4 depicts a representative architecture for the neural network model (multi-layer) with an input layer, a hidden layer and an output layer, trained and tested by a Back-propagation algorithm (BPNN). The ANN is “fed” randomly with the database and calculates the error between the experimental and predicted values. Then, it back propagates changing the weights and biases to obtain the smallest error. For all the models, the input variables chosen from the entire database were four maternal parameters: Age, p-BMI, weight status (normal weight, overweight, or obese) and GA at which maternal blood sample was taken. The output maternal variable was one biomarker: leptin, adiponectin, resistin, 8-oxodG, MDA or CP concentrations at the third trimester of pregnancy (input and output variables are depicted in Table 2).

#### 4.6.1. ANN Model

The experimental database (*n* =  68) was randomly divided into learning (79%) and validation (21%) and then, the input variables were normalized in the range of 0.1 to 0.9, as previously described [29]. The output variable was not normalized. 

Each neuron (*n*) has weights (*Wi* and *Wo*) and biases (*b*1 and *b*2) in the hidden and output layers (1) and (2):

*n*_1_ = *Wi* × *In*_1_ + *Wi* × *In*_2_ + …… + *Wi* × *In_k_* + *b*1 (1)

where *In* is the input variable. The value of each neuron is the argument of the transfer functions (*f* and *g*):

Adipokine or oxidative stress marker (*output*) = *g* (*Wo* × *f* (*Wi* × *In* + *b*1) + *b*2) (2)

where *f* is a hyperbolic tangent transfer function (TANSIG) and *g* is a linear transfer function (PURELIN) or Log-Sigmoid function (LOGSIG).

We applied different transfer functions to obtain the best performance for the models. As a result of the ANN model, Equation (2) with TANSIG-PURELIN was Equation (3):

(3) $$output=\sum_{s}\left\{Wo_{\left(l,s\right)}\cdot \left[\frac{2}{1+e^{-2\cdot \left(\sum_{k}Wi_{\left(s,k\right)}\cdot In_{k}+b1_{\left(s,1\right)}\right)}}-1\right]\right\}+b2_{\left(l,1\right)}$$

For other ANN models, Equation (2) considering TANSIG-LOGSIG was Equation (4), where *n_output_* is:

(4) $$\begin{array}{l} output=\frac{1}{\left(1+\exp\left(-n_{output\_layer}\right)\right)}\\ n_{output\_layer}=\sum_{s}\left\{Wo_{\left(l,s\right)}\cdot \left[\frac{2}{1+e^{-2\cdot \left(\sum_{k}Wi_{\left(s,k\right)}\cdot In_{k}+b1_{\left(s,1\right)}\right)}}-1\right]\right\}+b2_{\left(l,1\right)} \end{array}$$

#### 4.6.2. ANN Learning

In this work, to change the weights and biases, we applied the Levenberg–Marquardt (LM) algorithm, following our previously reported methods [29]. This uses the adaptation as follows:

(5) $$\Delta w={\left(J^{T}J+\mu I\right)}^{-1}J^{T}e$$

where *J* is the Jacobian matrix (first derivative); *e* is a vector of network errors; *μ* is the combination coefficient with a value of 0.001 and *I* is the identity matrix.

The root mean square error (RMSE) was applied as the error function which describes the performance of the network according to the following Equation (6):

(6) $$RMSE=\sqrt{\frac{\left(\sum_{q=1}^{Q}{\left(y_{q,\exp}-y_{q,ANNsim}\right)}^{2}\right)}{Q}}$$

where *Q* is the number of data points (*n* = 68); $y_{q,\exp}$ is the experimental data and $y_{q,ANNsim}$ is the network prediction.

#### 4.6.3. Results for Maternal Adipokines and Oxidative Stress Marker ANN Models

The proposed ANN models for MDA and CP followed Equation (7) with TANSIG-PURELIN:

(7) $$MDA\left(or\right)CP\_concentration=\sum_{s=1}^{S}\left[Wo_{\left(1,s\right)}\left(\frac{2}{1+\exp\left(-2\left(\sum_{k=1}^{K}\left(Wi_{\left(s,k\right)}In_{\left(k\right)}\right)+b1_{\left(s\right)}\right)\right)}-1\right)\right]+b2_{\left(1\right)}$$

Equation (8) gives adiponectin, leptin, resistin and 8-oxodG with TANSIG-LOGSIG, where *n_output_* is:

(8) $$\begin{array}{l} adiponectin\_or\_resistin\_or\_leptin\_or8-oxodG=\frac{1}{\left(1+\exp\left(-n_{output\_layer}\right)\right)}\\ n_{output\_layer}=\sum_{s}\left\{Wo_{\left(l,s\right)}\cdot \left[\frac{2}{1+e^{-2\cdot \left(\sum_{k}Wi_{\left(s,k\right)}\cdot In_{k}+b1_{\left(s,1\right)}\right)}}-1\right]\right\}+b2_{\left(l,1\right)} \end{array}$$

Equations (7) and (8) give the maternal adipokine or oxidative stress marker concentrations with weights and biases in Table A1 (Adiponectin ANN model, 4-8-1), Table A2 (Leptin ANN model, 4-8-1), Table A3 (Resistin ANN model, 4-9-1), Table A4 (carbonylated proteins ANN model, 4-8-1), Table A5 (MDA ANN model, 4-6-1) and Table A6 (8-oxodG ANN model, 4-9-1) in Appendix A.

#### 4.6.4. Statistical Tests for ANN Model Validation

ANN model validation was performed using linear regression models of the experimentally measured adipokines and oxidative stress marker concentrations versus the simulated ones (learning and validation database), obtaining the slope and intercept (Figure 3). Then, we applied a statistical test (slope and intercept, [59]) in which the upper and lower intervals of the slope and intercept must be near 1.0 and 0 respectively, with a 99.8% confidence level according to the Student *t*-test. 

The regression coefficient (R^2^) was then obtained from linear regression models for each biochemical value:

$$\left(Adipokine_{sim\;}or\;Redox\;marker_{sim}=a+b\;Adipokine\;or\;Redox\;marker_{exp}\right)$$

### 4.7. Sensitivity Analysis

To obtain the relative biological importance of maternal variables in predicting adipokine and oxidative stress marker concentrations, we performed a sensitivity analysis, as proposed by [33], based on the partitioning of connection weights:

(9) $$I_{j}=\frac{\sum_{m=1}^{Nh}\left(\left(\frac{\left|W_{jm}^{ih}\right|}{\sum_{k=1}^{Ni}\left|W_{km}^{ih}\right|}\right)\times \left|W_{mn}^{ho}\right|\right)}{\sum_{k=1}^{Ni}\left\{\sum_{m=1}^{Nh}\left(\frac{\left|W_{km}^{ih}\right|}{\sum_{k=1}^{Ni}\left|W_{km}^{ih}\right|}\right)\times \left|W_{mn}^{ho}\right|\right\}}$$

where $I_{j}$ is the relative importance of the input variable on the output variable; $N_{i}$ is the number of input neurons; $N_{h}$ is the number of hidden neurons; W is the connection weight; and the superscripts i,h and o refer to input, hidden and output layer.

## 5. Conclusions

The ANN models accurately predicted adipokine and oxidative stress marker concentrations in the third trimester of pregnancy based on feasible and easy to measure clinical and anthropometric variables, allowing to obtain the reference blood concentrations in pregnant women with or without pre-pregnancy overweight and obesity. The early prediction of alterations in these markers (prenatal) could be used by clinicians to implement strategies that improve metabolic and nutrition status, influencing perinatal outcomes in overweight/obese women. The prediction of these maternal biomarkers adds quantitative dimensions to the assessment of pregnancy follow-up, which could particularly benefit the group of patients with normal pregnancy outcomes despite abnormal adipokine and oxidative stress marker concentrations. Alterations in these markers may modify nutrient utilization by the fetus, and thus, impact fetal growth. Being large for gestational age or macrosomic at birth is associated with higher adiposity later in life. Consequently, the prediction of these alterations early in pregnancy may guide clinicians in selecting different strategies to improve nutrition and monitor fetal growth closely. Studies are in progress to evaluate if the models may be generalized to other settings.

## Figures and Tables

**Table 1:** Clinical characteristics and biochemical data in peripheral maternal blood classified according to pre-gestational BMI.

Variables	Normal (*n* = 25)	Overweight (*n* = 21)	Obese (*n* = 22)
Age (years)	27.9 ± 1.90	30.3 ± 1.90	32.9 ± 1.40 ^b^
p-BMI (kg/m^2^)	22.0 ± 0.30	27.6 ± 0.30 ^a^	35.1 ± 1.00 ^b,c^
GA (weeks)	34.4 ± 0.70	35.6 ± 0.60	35.8 ± 0.70
Glucose (mg/dL)	80.0 ± 2.4	89.4 ± 4.60	87.3 ± 4.10
Insulin (μIU/mL)	7.80 ± 2.0	10.6 ± 1.70	14.9 ± 3.80
HOMA-IR	1.60 ± 0.50	2.60 ± 0.70	3.80 ± 1.20
Leptin (ng/mL)	24.3 ± 3.50	39.7 ± 5.60	41.2 ± 10.7
Adiponectin (μg/mL)	16.2 ± 2.30	12.3 ± 2.10	9.20 ± 1.90 ^b^
Resistin (ng/mL)	23.0 ± 3.00	20.1 ± 4.20	13.4 ± 2.20 ^b^
MDA (nmol/mg dry weight)	0.12 ± 0.006	0.13 ± 0.01	0.22 ± 0.02 ^b,c^
CP (nmol/mg protein)	10.0 ± 0.40	10.9 ± 0.70	16.7 ± 1.10 ^b,c^
8-oxodG (ng/mL)	218 ± 18.4	198 ± 8.90	188 ± 4.30

p-BMI: pregestational body mass index; GA: gestational age; MDA: malondialdehyde; CP: carbonylated proteins; 8-oxodG: 8-hydroxy-2′-deoxyguanosine. Values represent mean ± SEM (Standard Error of the Mean). *p* values were estimated using one-way ANOVA with DMS post hoc test. ^a^ *p* < 0.05 overweight versus normal; ^b^ *p* < 0.05 obese versus normal; ^c^ *p* < 0.05 obese versus overweight.

**Table 2:** Input (clinical and anthropometric variables) and output (biochemical values) range conditions in the maternal ANN model.

Input Variables	Range	Output Variables	Range
Age (years)	14–43	Leptin (ng/mL)	0.38–223.4
p-BMI (kg/m^2^)	18.6–48.3	Adiponectin (μg/mL)	4.1–40.4
GA (weeks)	28.3–40.4	Resistin (ng/mL)	0.7–90.4
Weight Status Classification	Normal, overweight or obese	MDA (nmol/mg dry weight)	0.04–0.55
		8-oxodG (ng/mL)	160–642
		CP (nmol/mg protein)	5.72–30.5

p-BMI: pregestational body mass index; GA: gestational age; MDA: malondialdehyde; CP: carbonylated proteins; 8-oxodG: 8-hydroxy-2′-deoxyguanosine.

## Abbreviations

ANN	Artificial neural network
p-BMI	Pre-pregnancy Body Mass Index
CP	Carbonylated proteins
8-oxodG	oxidized base 8-oxo-2′-deoxyguanosine
MDA	Malondialdehyde
GA	Gestational age
CP	Carbonylated proteins
MA	Maternal age

## Appendix A

Weight and biases for ANN models.

**Table A1:** Weights and biases for the ANN model predicting adiponectin concentration.

8 Neurons on Hidden Layer (*k* = 8 and *l* = 1)								
*Wi*_{*s*,*k*}_	*Wi*_{*s*,1}_	*Wi*_{*s*,2}_	*Wi*_{*s*,3}_	*Wi*_{*s*,4}_				
	−0.052517	3.7407	0.30914	−0.83713				
	−28.294	−6.5884	1.4140	11.300				
	−6.9433	4.7470	−20.738	2.7172				
	−1.4014	−1.2418	2.5042	2.3499				
	12.935	−11.478	7.3280	6.8132				
	3.3992	6.2892	−2.5518	−4.9357				
	9.3624	−10.582	4.9177	6.7825				
	4.0123	27.372	10.360	3.5215				
*Wo*_{*l*,*s*}_	*Wo*_{1,1}_	*Wo*_{1,2}_	*Wo*_{1,3}_	*Wo*_{1,4}_	*Wo*_{1,5}_	*Wo*_{1,6}_	*Wo*_{1,7}_	*Wo*_{1,8}_
	4.0988	10.884	−0.96316	−5.4384	−6.5596	−2.8140	7.3260	−13.678
*b*1_{*s*,1}_	*b*1_{5,1}_							
	−0.45666							
	24.442							
	12.415							
	−1.3457							
	−6.3817							
	−1.6500							
	−4.2754							
	−7.4087							
*b*2_{*l*,*s*}_	*b*2_{1,1}_							
	−0.54830							

**Table A2:** Weights and biases for the ANN model predicting leptin concentration.

8 Neurons on Hidden Layer (*k* = 8 and *l* = 1)								
*Wi*_{*s*,*k*}_	*Wi*_{*s*,1}_	*Wi*_{*s*,2}_	*Wi*_{*s*,3}_	*Wi*_{*s*,4}_				
	−2.1829	0.79377	−8.8924	−4.8956				
	13.941	7.3074	9.7328	−0.5.0633				
	12.187	−14.506	−16.893	−12.973				
	47.113	−32.460	−16.992	−19.329				
	−18.396	7.7265	5.3903	−15.068				
	−19.657	−21.542	10.787	14.645				
	−16.118	26.497	−1.4738	2.4320				
	0.17226	−3.3014	7.6009	6.3612				
*Wo*_{*l*,*s*}_	*Wo*_{1,1}_	*Wo*_{1,2}_	*Wo*_{1,3}_	*Wo*_{1,4}_	*Wo*_{1,5}_	*Wo*_{1,6}_	*Wo*_{1,7}_	*Wo*_{1,8}_
	−2.2859	2.1313	1.0830	−0.93728	−2.0859	4.1075	0.46330	−2.5751
*b*1_{*s*,1}_	*b*1_{5,1}_							
	7.0867							
	−22.895							
	19.031							
	10.810							
	−2.7510							
	−2.7589							
	6.3961							
	−5.1093							
*b*2_{*l*,*s*}_	*b*2_{1,1}_							
	2.2934							

**Table A3:** Weights and biases for the ANN model predicting resistin concentration.

9 Neurons on Hidden Layer (*k* = 9 and *l* = 1)									
*Wi*_{*s*,*k*}_	*Wi*_{*s*,1}_	*Wi*_{*s*,2}_	*Wi*_{*s*,3}_	*Wi*_{*s*,4}_					
	−0.12216	−6.136	2.6049	9.6953					
	27.276	11.076	−2.3404	−5.9354					
	−0.49141	−1.0027	1.6407	−0.2277					
	16.723	−1.2056	16.100	−3.8005					
	12.611	8.6390	−1.4910	8.5175					
	−0.69780	2.7485	−60.73	1.1423					
	−0.11218	9.3635	−14.79	6.7889					
	−8.4773	2.8179	−10.031	0.56276					
	3.6918	1.3614	9.154	−5.7361					
*Wo*_{*l*,*s*}_	*Wo*_{1,1}_	*Wo*_{1,2}_	*Wo*_{1,3}_	*Wo*_{1,4}_	*Wo*_{1,5}_	*Wo*_{1,6}_	*Wo*_{1,7}_	*Wo*_{1,8}_	*Wo*_{1,9}_
	7.5398	12.500	6.2809	−5.7172	9.6729	10.140	−0.10398	−6.2219	6.4819
*b*1_{*s*,1}_	*b*1_{5,1}_								
	0.5.1064								
	−24.66								
	0.68847								
	−12.01								
	−0.0111								
	1.0737								
	−9.0268								
	6.8763								
	−2.4130								
*b*2_{*l*,*s*}_	*b*2_{1,1}_								
	0.81976								

**Table A4:** Weights and biases for the ANN model predicting carbonylated proteins concentration.

8 Neurons on Hidden Layer (*k* = 8 and *l* = 1)								
*Wi*_{*s*,*k*}_	*Wi*_{*s*,1}_	*Wi*_{*s*,2}_	*Wi*_{*s*,3}_	*Wi*_{*s*,4}_				
	−3.4952	−24.375	−4.9253	−5.3093				
	−14.758	−2.6467	−8.7481	3.5794				
	2.0876	−11.504	5.7321	−0.96934				
	−0.67465	−5.0937	−3.3881	2.6267				
	−28.741	−10.542	14.773	31.069				
	7.6510	−25.181	9.7527	−0.41751				
	−5.0850	−1.6182	−3.9322	0.55163				
	−33.142	−33.116	12.653	33.422				
*Wo*_{*l*,*s*}_	*Wo*_{1,1}_	*Wo*_{1,2}_	*Wo*_{1,3}_	*Wo*_{1,4}_	*Wo*_{1,5}_	*Wo*_{1,6}_	*Wo*_{1,7}_	*Wo*_{1,8}_
	5.5379	9.0540	−35.747	−37.903	−33.806	32.181	−10.788	36.050
*b*1_{*s*,1}_	*b*1_{5,1}_							
	3.7180							
	9.8466							
	1.5882							
	9.3072							
	−12.59							
	2.2808							
	4.2440							
	−2.7282							
*b*2_{*l*,*s*}_	*b*2_{1,1}_							
	15.546							

**Table A5:** Weights and biases for the ANN model predicting MDA concentration.

6 Neurons on Hidden Layer (*k* = 6 and *l* = 1)						
*Wi*_{*s*,*k*}_	*Wi*_{*s*,1}_	*Wi*_{*s*,2}_	*Wi*_{*s*,3}_	*Wi*_{*s*,4}_		
	−12.646	5.3087	30.714	−3.5685		
	−7.3917	22.045	−16.992	12.924		
	7.7847	−4.6160	7.8948	2.3106		
	−7.2892	21.445	−17.128	13.605		
	0.33297	22.854	−20.959	−4.7038		
	2.8826	−1.1998	4.1718	0.65482		
*Wo*_{*l*,*s*}_	*Wo*_{1,1}_	*Wo*_{1,2}_	*Wo*_{1,3}_	*Wo*_{1,4}_	*Wo*_{1,5}_	*Wo*_{1,6}_
	0.07501	−3.1628	0.30868	3.1815	0.06675	−1.7149
*b*1_{*s*,1}_	*b*1_{5,1}_					
	−14.317					
	−5.8818					
	−11.299					
	−5.9995					
	5.4172					
	−6.5706					
*b*2_{*l*,*s*}_	*b*2_{1,1}_					
	−1.2413					

**Table A6:** Weights and biases for the ANN model predicting 8-oxodG concentration.

9 Neurons on Hidden Layer (*k* = 9 and *l* = 1)									
*Wi*_{*s*,*k*}_	*Wi*_{*s*,1}_	*Wi*_{*s*,2}_	*Wi*_{*s*,3}_	*Wi*_{*s*,4}_					
	12.299	−5.2680	1.6350	−5.2381					
	−7.6752	0.57821	7.8892	−2.1634					
	1.1911	2.0545	0.74680	2.3719					
	14.498	−14.829	1.8490	−6.7092					
	−1.3056	−0.94936	−0.54253	−8.9123					
	−14.124	−3.1471	−33.748	−1.0474					
	7.3859	−3.0612	−1.4795	6.7889					
	14.169	10.927	−8.5031	−5.7630					
	−6.0628	7.4203	9.4	−6.8565					
*Wo*_{*l*,*s*}_	*Wo*_{1,1}_	*Wo*_{1,2}_	*Wo*_{1,3}_	*Wo*_{1,4}_	*Wo*_{1,5}_	*Wo*_{1,6}_	*Wo*_{1,7}_	*Wo*_{1,8}_	*Wo*_{1,9}_
	−5.7998	−11.464	−0.59086	−0.21468	0.18834	−0.50880	73.576	−4.9478	7.7960
*b*1_{*s*,1}_	*b*1_{5,1}_								
	10.626								
	3.6306								
	−3.0887								
	2.7216								
	5.3955								
	12.883								
	1.1882								
	−6.1366								
	−2.4130								
*b*2_{*l*,*s*}_	*b*2_{1,1}_								
	−6.9216								

**Table A7:** Slope and intercept values for adipokines statistical test.

Adiponectin		Leptin		Resistin	
*a*_lower_	*a*_upper_	*a*_lower_	*a*_upper_	*a*_lower_	*a*_upper_
−0.0189	0.0546	−0.0124	0.0378	−0.0200	0.0531
*b*_lower_	*b*_upper_	*b*_lower_	*b*_upper_	*b*_lower_	*b*_upper_
0.8270	1.0287	0.8558	1.0535	0.8027	1.0480

**Table A8:** Slope and intercept values for oxidative stress markers statistical test.

Carbonylated Proteins		MDA		8-oxodG	
*a*_lower_	*a*_upper_	*a*_lower_	*a*_upper_	*a*_lower_	*a*_upper_
−1.1971	1.3036	−0.0075	0.0314	−0.0159	0.0254
*b*_lower_	*b*_upper_	*b*_lower_	*b*_upper_	*b*_lower_	*b*_upper_
0.8289	1.0916	0.8158	1.0367	0.8677	1.0760
